# Evaluation of organizational and social commitments and related factors during the coronavirus pandemic of healthcare workers in northern Iran

**DOI:** 10.1186/s12992-020-00658-0

**Published:** 2021-01-19

**Authors:** Zahra Aghalari, Hans-Uwe Dahms, Somayeh Jafarian, Hemmat Gholinia

**Affiliations:** 1 Environmental Health Engineer, Social Determinants of Health Research Center, Health Research Institute, Babol University of Medical Sciences, Babol, Iran; 2 Department of Biomedical Science and Environmental Biology, College of Life Science, Kaohsiung Medical University, Kaohsiung, Taiwan; 3 Research Center for Environmental Medicine, KMU - Kaohsiung Medical University, Kaohsiung, 80708 Taiwan; 4 Health Services Management, B.A. in Environmental Health Engineering, Health Department, Social Determinants of Health Research Center, Health Research Institute, Babol University of Medical Sciences, Babol, Iran; 5 Health Research Institute, Babol University of Medical Sciences, Babol, Iran

**Keywords:** Coronavirus, Organizational commitments, Social commitments, Healthcare workers

## Abstract

**Background:**

Serious conditions caused by the coronavirus epidemic are expected to affect the mental and physical health, organizational and social commitments of healthcare workers. Therefore, this study aimed to evaluate the organizational and social commitments and related factors during the coronavirus pandemic of healthcare workers in northern Iran.

**Methods:**

This descriptive-analytical study was conducted in 2020 among 260 healthcare workers of Babol health centers by a stratified-random sampling method. Data were collected according to a valid and reliable questionnaire consisting of three parts: 8 questions about personal and job characteristics, 15 questions from Porter Organizational Commitment Questionnaire (OCQ), 15 questions from Carroll’s social responsibility. Each question was scored on the Likert scale of organizational and social commitment questionnaires. Data were analyzed by chi-square and logistic regression. *P* < 0.05 was considered as statistically significant.

**Results:**

None of the healthcare workers belonged to the category of low organizational commitments. A portion of 27.7% of the healthcare workers had moderate organizational commitments and 72.3% had high organizational commitments. A portion of 9.2% of the healthcare workers had moderate social commitments and 90.8% had high social commitments. Chi-square showed that education (*p* = 0.001), job position (*p =* 0.001) and the area in which healthcare workers were present for service (*p* = 0.002) were significantly associated with organizational commitments. According to OR in the logistic regression model, healthcare workers with master’s and doctoral education levels had 3.482 times more social commitments than others and the health group had 2.455 times more social commitments compared to the treatment group.

**Conclusion:**

The results of this study showed that at the time of the coronavirus outbreak, the healthcare workers in Babol had very positive and high organizational and social commitments. As the world struggles with the coronavirus pandemic, employee and organizational productivity may decline due to the fear and anxiety of healthcare workers in various organizations. It is expected that managers of health-related organizations, social, economic, and cultural organizations use the results of this study to identify factors affecting the organizational and social commitments of employees and strengthen them.

## Background

Coronaviruses are a large family of viruses that may cause respiratory infections such as colds and more severe illnesses such as the Middle East Respiratory Syndrome (MERS) [1, 2]. The outbreak of coronavirus started in December 2019 in Wuhan, China and subsequently spread from there to other countries. Many regions of the world, including Africa, the Americas, the Eastern Mediterranean, Europe, Southeast Asia, and the Western Pacific, became involved in the fight against COVID-19 [3]. Coronavirus also spread in Iran and quickly affected various economic, social, cultural, and health activities overthere. In the coronavirus pandemic in Iran, part of the screening, identification, and treatment of patients was the responsibility of healthcare workers and they were providing services around the clock to control the disease. Bad conditions caused by the coronavirus epidemic are expected to affect the mental and physical health, as well as the organizational and social commitments of healthcare workers [4, 5].

Organizational commitment is considered as an emotional and psychological dependence on the organization, according to which a person who is strongly committed and engages in it and enjoys membership in the organization [6]. Organizational commitment leads to loyalty to the organization and greater knowledge of it [7]. According to Cohen, organizational commitment increases performance effectiveness, productivity, and reduces the tendency to leave service and commitment at the individual and organizational levels. Committed people use all their power to advance their goals and the goals of the organization and do not limit themselves to do things within the framework of existing laws and structures. Existence of non-committed staff causes a quantitative and qualitative decline in the performance of the organization and leads to a lack of motivation to provide services [8, 9]. During the coronavirus pandemic, a number of healthcare workers refused to provide services and attendance at their workplaces, including hospitals and health centers, and forgot about their organizational commitments.

Social commitments or social responsibilities are among the most important elements of organizations [10]. Observance of social commitments by employees promotes organizational performance and commitment and increases client satisfaction [11]. Social commitments are defined as a set of activities that the owners of capital and economic enterprises perform as an effective and beneficial factor in society [12].

The health sector in the community strives to provide modern health and treatment services to the community and to accept its social responsibility [13]. The main question is what percentage of healthcare workers adhere to social commitments and do not refuse to provide services in the event of epidemics and pandemics. Healthcare workers should feel responsible to their clients and patients in the event of natural disasters, pandemics and epidemics, and adhere to their organizational and social commitments. Several studies have been conducted on organizational and social commitments in clinical occupations such as physicians and nurses [14, 15], but no research has been done on organizational and social commitments during a particular disease pandemic, such as the coronavirus, among healthcare workers. Therefore, this study aimed to evaluate the organizational and social commitments and related factors during the coronavirus pandemic of healthcare workers in northern Iran.

## Methods

This is a descriptive-analytical study that was conducted in 2020 among healthcare workers in Babol. Healthcare workers included: physicians, midwives, experts from public health, environmental and occupational health, as well as laboratory technicians, receptionists and behvarz (Iranian rural health workers), who were divided into two groups, treatment groups including: physicians, midwives and laboratory technicians, and others in the health group.

In this study, the sample size was estimated by examining internal and external studies and Cochran’s formula, taking into account the 95% confidence interval, 50% prevalence estimate, 0.05 error rate, and a population size of 800 people, and 260 healthcare workers. Samples were selected by the stratified-random method. Healthcare workers were selected for this study due to their willingness to answer the questionnaire, had 1 year of work experience, and were present in the workplace during the coronavirus pandemic.

The data collection tools were a questionnaire consisting of three parts:

1. 8 questions about personal and occupational characteristics and 4 questions about a person’s condition during a coronavirus epidemic.
2. 15 questions were reported from Porter Organizational Commitment Questionnaire (OCQ): Validity of this questionnaire in different studies [16, 17] for the general organizational commitment, emotional commitment, continuous commitment and excitement commitment respectively 0.88, 0.77, 0.61 and 0.79 and its reliability through Cronbach’s alpha 0.91. This questionnaire was evaluated with a 7-point Likert scale (strongly disagree, disagree, slightly disagree, neither agree nor disagree, slightly agree, agree, strongly agree). The minimum score of this questionnaire was 15 and the maximum score was 105. Whose mean score was 35 were in the group of low organizational commitments, 36 to 70 were in the group of medium organizational commitments and 71 to 105 were in the group of high organizational commitments.
3. 15 questions from Carroll’s social responsibility (CSR): The original Carroll’s social responsibility (1991) has 35 questions. However, in this study only 15 questions were used that were compatible with the coronavirus pandemic conditions. In this questionnaire, questions related to the environment and organizational regulations were removed [10, 18]. The content validity of this questionnaire was confirmed and its reliability was 0.95 [19]. This questionnaire was evaluated with a five-point Likert scale (very low = 1, low = 2, medium = 3, high = 4, very high = 5). The minimum score of this questionnaire was 15 and the maximum score was 75. Whose mean score was 25 were in the low social commitment group, 26 to 50 were in the moderate social commitment group, and 51 to 75 were in the good social commitment group.

To observe ethical considerations, the purpose of the study was explained to all of the healthcare workers and their consent was obtained prior to administering the questionnaires. It was ensured that the questionnaires would be used in general and without mentioning the name.

Questionnaires were completed self-reportedly by the staff of the centers. Data were analyzed by chi-square and logistic regression using SPSS 22.

## Results

The mean age of 260 participants in this study was 40.35 ± 8.55 years, the minimum and maximum ages were 22 and 60 years, respectively. Most of the healthcare workers were women (64.6%) and 52.3% had an academic degree and were either bachelor, master and doctor. 57.3% were in the health group and 42.7% were in the treatment group. The mean work experience of the healthcare workers were 15.09 ± 8.53 years. A percentage of 71.2% of healthcare workers were working in rural areas and 28.8% in urban areas. 16.5% of healthcare workers became infected with Coronavirus during their service. 36.9% of healthcare workers were not satisfied with their presence at work during the outbreak of coronavirus.

The mean scores of the organizational commitment questionnaire of the healthcare workers were 77.80 ± 11.66, the lowest and highest scores were 45 and 99, respectively. None of the healthcare workers fall in the category of low organizational commitments. A number of 27 healthcare workers (27.7%) had medium organizational commitments and 188 of healthcare workers (72.3%) had high organizational commitments. The mean of the highest scores in the organizational commitment questionnaire was related to question 4 regarding satisfaction with continuing to work in the health organization (6.24 ± 1.27), question 6 regarding honor to be present in the health organization (6.20 ± 1.25) (Table 1).

**Table 1:** Frequency of responses to organizational commitments of healthcare workers in Babol-2020

Questions based on the condition of individuals during the coronavirus pandemic	Response Number (percentage)							Mean ± SD
	Strongly disagree	Disagree	Slightly disagree	Neither agree nor disagree	Slightly agree	Agree	Strongly agree	
1. During the coronavirus pandemic, I wanted to work much harder for this organization than I usually expected.	8 (3.1)	7 (2.7)	10 (3.8)	21 (8.1)	34 (13.1)	63 (24.2)	117 (45)	5.78 ± 1.55
2. During the coronavirus pandemic, I used to recommend my workplace organization to my friends as the best organization.	10 (3.8)	8 (3.1)	10 (3.8)	38 (14.6)	38 (14.6)	64 (24.6)	92 (35.4)	5.48 ± 1.62
3. During the coronavirus pandemic, I felt very loyal to my workplace organization.	21 (8.1)	13 (5)	4 (1.5)	14 (5.4)	13 (5)	30 (11.5)	165 (63.5)	5.82 ± 1.96
4. During the coronavirus pandemic, to continue working for this organization, I accepted any kind of task.	3 (1.2)	4 (1.5)	6 (2.3)	16 (6.2)	18 (6.9)	51 (19.6)	162 (62.3)	6.24 ± 1.27
5. During the coronavirus pandemic, I thought my values and the values of the organization were very similar.	13 (5)	9 (3.5)	8 (3.1)	35 (13.5)	31 (11.9)	60 (23.1)	104 (40)	5.53 ± 1.71
6. During the coronavirus pandemic, I was proud to tell others that I was a member of this organization.	4 (1.5)	1 (0.4)	3 (1.2)	23 (8.8)	26 (10)	45 (17.3)	158 (60.8)	6.20 ± 1.25
7. During the coronavirus pandemic, I thought I could not work as well in another organization.	85 (32.7)	63 (24.2)	25 (9.6)	55 (21.2)	12 (4.6)	11 (4.2)	9 (3.5)	2.67 ± 1.66
8. During the coronavirus pandemic, I thought my career success came from this organization.	13 (5)	10 (3.8)	22 (8.5)	52 (20)	35 (13.5)	63 (24.2)	65 (25)	5.05 ± 1.71
9. During the coronavirus pandemic, I thought leaving this organization would make very little change in my current situation.	51 (19.6)	30 (11.5)	18 (6.9)	88 (33.8)	27 (10.4)	20 (7.7)	26 (10)	3.66 ± 1.87
10. During the coronavirus pandemic, I was happy to choose this organization to work from among other organizations.	10 (3.8)	7 (2.7)	13 (5)	40 (15.4)	35 (13.5)	69 (26.5)	86 (33.1)	5.43 ± 1.61
11. During the coronavirus pandemic, I thought staying in this organization would have many benefits for me.	17 (6.5)	24 (9.2)	20 (7.7)	44 (16.9)	25 (9.6)	39 (15)	91 (35)	4.98 ± 1.97
12. During the coronavirus pandemic, I often agreed with the organization’s rules on staff affairs.	24 (9.2)	29 (11.2)	30 (11.5)	58 (22.3)	27 (10.4)	32 (12.3)	60 (23.1)	4.42 ± 1.97
13. During the coronavirus pandemic, I was sensitive to the fate of this organization.	10 (3.8)	2 (0.8)	8 (3.1)	40 (15.4)	31 (11.9)	60 (23.1)	109 (49.1)	5.67 ± 1.55
14. During the coronavirus pandemic, I was sensitive about working in this organization.	28 (10.8)	12 (4.6)	23 (8.8)	43 (16.5)	26 (10)	34 (13.1)	94 (36.2)	4.94 ± 2.06
15. During the coronavirus pandemic, I came to the conclusion that the decision to work for this organization was constructive for me.	8 (3.1)	8 (3.1)	15 (5.8)	27 (10.4)	19 (7.3)	29 (11.2)	154 (59.2)	5.86 ± 1.68

Chi-square indicated that education was significantly related to organizational commitments (*p =* 0.001), so that healthcare workers with diploma and bachelor had higher organizational commitments. Job position was significantly related to organizational commitments (*p* = 0.001) so that health group had more organizational commitments. Healthcare workers serving in rural areas had higher organizational commitments (*p* = 0.002) (Table 2).

**Table 2:** Relationship between different variables and organizational commitments of healthcare workers in Babol-2020

Variables	Levels	Medium organizational commitments Number (percentage)	High organizational commitments Number (percentage)	χ^2^	*P*-value
Sex	Female	52 (31)	116 (69)	2.520	0.112
	Male	20 (21.7)	72 (78.3)		
Education	Diploma	16 (16.8)	79 (83.2)	19.745	0.001
	Above diploma	11 (37.9)	18 (62.1)		
	Bachelor	24 (25)	72 (75)		
	Masters and PhD	21 (52.5)	19 (47.5)		
Age	20–30	9 (22.5)	31 (77.5)	0.781	0.677
	31–50	55 (29.1)	134 (70.9)		
	Over 51	8 (25.8)	23 (74.2)		
Work experience	1–10	34 (35.1)	63 (64.9)	4.480	0.110
	11–20	18 (21.7)	65 (78.3)		
	Over 21	20 (25)	60 (75)		
Groups	Health group	25 (16.8)	124 (83.2)	20.761	0.001
	Treatment group	47 (42.3)	64 (57.7)		
Coronavirus infection during service	Yes	13 (30.2)	30 (69.8)	0.166	0.684
	No	59 (27.2)	158 (72.8)		
Service location	Urban	31 (41.3)	44 (58.7)	9.795	0.002
	rural	41 (22.2)	144 (77.8)		
Family consent to attend work	Yes	37 (22.6)	127 (77.4)	5.841	0.016
	No	35 (36.5)	61 (63.5)		

The mean scores of the social commitment questionnaire of healthcare workers were 61.12 ± 8.03, the lowest and highest scores were 35 and 75, respectively. None of the healthcare workers fell into the category of low social commitments. 24 healthcare workers (9.2%) were in the medium social commitment group and 236 healthcare workers (90.8%) were in the high social commitment group. The mean of the highest scores in the social commitment questionnaire related to question 2 regarding the right of people to have adequate health care services (4.68 ± 0.63), question 12 regarding happiness in solving people’s problems during the coronavirus (altruism) (4.78 ± 0.78) (Table 3).

**Table 3:** Frequency of responses to social commitment of healthcare workers in Babol-2020

Questions based on the condition of individuals during the coronavirus pandemic	Response Number (percentage)					Mean ± SD
	Strongly disagree	Disagree	Neither agree nor disagree	Agree	Strongly agree	
1. During the coronavirus pandemic, customer satisfaction was very important to me.	5 (1.9)	1 (0.4)	10 (3.8)	68 (26.2)	176 (67.2)	4.57 ± 0.76
2. During the coronavirus pandemic, I was very much in favor of the phrase, “People have the right to receive adequate health care.”	2 (0.8)	2 (0.8)	6 (2.3)	56 (21.5)	194 (74.6)	4.68 ± 0.63
3. During the coronavirus pandemic, I involved myself in people’s problems.	8 (3.1)	6 (2.3)	7 (2.7)	62 (23.8)	177 (68.1)	4.51 ± 0.90
4. During the coronavirus pandemic, I did not pay much attention to the profitability of the organization.	32 (12.3)	26 (10)	41 (15.8)	45 (17.3)	116 (44.6)	3.71 ± 1.42
5. During the coronavirus pandemic, monetary or non-monetary rewards did not matter to me.	37 (14.2)	66 (25.4)	43 (16.5)	53 (20.4)	61 (23.5)	3.13 ± 1.39
6. During the coronavirus pandemic, I was doing my job.	20 (7.7)	36 (13.8)	30 (11.5)	61 (23.5)	113 (43.5)	3.81 ± 1.32
7. During the coronavirus pandemic, in my opinion, paying attention to the interests of the healthcare staff was not the main goal of the Ministry of Health.	42 (16.2)	36 (13.8)	57 (21.9)	53 (20.4)	72 (27.7)	3.29 ± 1.41
8. During the coronavirus, Client satisfaction was important to me.	7 (2.7)	6 (2.3)	7 (2.7)	74 (28.5)	166 (63.8)	4.48 ± 0.87
9. During the coronavirus pandemic, I thought people were more important to me if I had to choose between the organization and the people.	15 (5.8)	10 (3.8)	63 (24.2)	73 (28.1)	99 (38.1)	3.88 ± 1.13
10. During the coronavirus pandemic, I was thinking of others.	5 (1.9)	8 (3.1)	14 (5.4)	55 (21.2)	178 (68.5)	4.51 ± 0.88
11. During the coronavirus pandemic, I accepted doing good to others.	10 (3.8)	11 (4.2)	26 (10)	51 (19.6)	162 (62.3)	4.32 ± 1.06
12. During the coronavirus pandemic, I was happy to be able to solve people’s problems.	4 (1.5)	6 (2.3)	8 (3.1)	45 (17.3)	197 (75.8)	4.63 ± 0.78
13. During the coronavirus pandemic, sometimes I did not pretend to advance my goals.	18 (6.9)	29 (11.2)	46 (17.7)	72 (27.7)	95 (36.5)	3.75 ± 1.24
14. During the coronavirus pandemic, I believed that “helping one’s fellow man is like worshiping God.”	4 (1.5)	6 (2.3)	8 (3.1)	57 (21.9)	185 (71.2)	4.58 ± 0.79
15. During the coronavirus pandemic, I enjoyed serving clients even without rewards	49 (18.8)	44 (16.9)	37 (14.2)	68 (26.2)	62 (23.8)	3.19 ± 1.45

Chi-square showed that at the time of the outbreak of the coronavirus pandemic, I did not have a significant relationship between age, sex, education, and work experience with social commitments (Table 4).

**Table 4:** Relationship between different variables and social commitments of healthcare workers in Babol-2020

Variables	Levels	Medium social commitments Number (percentage)	High social commitments Number (percentage)	χ^2^	*P*-value
Sex	Female	14 (8.3)	154 (91.7)	0.456	0.499
	Male	10 (10.9)	82 (89.1)		
Education	Diploma	10 (10.5)	85 (89.5)	1.090	0.779
	Above diploma	3 (10.3)	26 (89.7)		
	Bachelor	9 (9.4)	87 (90.6)		
	Masters and PhD	2 (5)	38 (95)		
Age	20–30	2 (5)	38 (95)	1.373	0.503
	31–50	18 (9.5)	171 (90.5)		
	Over 51	4 (12.9)	27 (87.1)		
Work experience	1–10	6 (6.2)	91 (93.8)	2.698	0.259
	11–20	11 (13.3)	72 (86.7)		
	Over 21	7 (8.8)	73 (91.2)		
Groups	Health group	11 (7.4)	138 (92.6)	1.423	0.233
	Treatment group	13 (11.7)	98 (88.3)		
Coronavirus infection during service	Yes	1 (2.3)	42 (97.7)	2.932	0.087
	No	23 (10.6)	194 (89.4)		
Service location	Urban	8 (10.7)	67 (89.3)	0.259	0.611
	rural	16 (8.6)	169 (91.4)		
Family consent to attend work	Yes	19 (11.6)	145 (88.4)	2.939	0.086
	No	5 (5.2)	91 (94.8)		

Multivariate logistic regression analysis revealed that education and occupation were independent predictors of organizational commitments. Healthcare workers with above diploma (OR = 0.320, 95% CI:0.117–0.873, *P* = 0.026), master and PhD (OR = 0.280, 95% CI: 0.107–0.735, *P* = 0.010) and in the health group (OR = 2.919, 95% CI: 1.450–5.878, *P* = 0.003) have higher organizational commitments. According to OR in the logistic regression, women had 1.247 times more organizational commitments than men, and the health group had 2.919 times more organizational commitments compared to the treatment group (Table 5).

**Table 5:** Predictors of organizational commitments of healthcare workers in Babol-2020

Variables	β	SE	Wald	P	OR	95% CI	
						Lower	Upper
Total organizational commitments							
Age_Cat			0.501	0.778			
31–50 years (Ref: 20–30)	−0.308	0.463	0.444	0.505	0.735	0.296	1.821
Over 51 years (Ref: 20–30)	−0.124	0.630	0.039	0.844	0.883	0.257	3.040
Female (Ref:Male)	0.221	0.353	0.392	0.531	1.247	0.625	2.489
Education			11.811	0.008			
Above diploma (Ref: Diploma)	−1.140	0.512	4.950	0.026	0.320	0.117	0.873
Bachelor (Ref: Diploma)	−0.171	0.421	0.166	0.684	0.843	0.369	1.923
Masters and PhD (Ref: Diploma)	−1.273	0.492	6.681	0.010	0.280	0.107	0.735
Groups (Ref: Treatment group)	1.071	0.357	8.997	0.003	2.919	1.450	5.878
Constant	1.334	0.584	5.221	0.022	3.798		

*SE* standard error, *OR* odds ratio, *CI* confidential interval, *Ref* reference

According to OR in the logistic regression model, healthcare workers with master’s and doctoral education levels had 3.482 times more social commitments than other and the health group had 2.455 times more social commitments compared to the treatment group (Table 6).

**Table 6:** Predictors of social commitments of healthcare workers in Babol-2020

Variables	β	SE	Wald	P	OR	95% CI	
						Lower	Upper
Total social commitments							
Age_Cat			1.195	0.550			
31–50 years (Ref: 20–30)	−0.829	0.825	1.010	0.315	0.437	0.087	2.199
Over 51 years(Ref: 20–30)	−0.982	0.954	1.060	0.303	0.375	0.058	2.429
Female (Ref:Male)	−0.735	0.499	2.172	0.141	0.480	0.180	1.274
Education			2.076	0.557			
Above diploma (Ref: Diploma)	0.111	0.736	0.023	0.881	1.117	0.264	4.724
Bachelor (Ref: Diploma)	0.219	0.562	0.151	0.697	1.244	0.414	3.742
Masters and PhD (Ref: Diploma)	1.248	0.874	2.037	0.154	3.482	0.628	19.319
Groups (Ref: Treatment group)	0.898	0.513	3.071	0.080	2.455	0.899	6.705
Constant	2.985	0.939	10.104	0.001	19.791		

*SE* standard error, *OR* odds ratio, *CI* confidential interval, *Ref*. reference

## Discussion

None of the healthcare workers was belonging to the category of low organizational commitments. A portion of 27.7% of healthcare workers had medium organizational commitments and 72.3% had high organizational commitments. The results of this section show that at the time of the coronavirus outbreak, the healthcare workers of health centers in Babol had positive organizational commitments. A study by Iravan Masoodi et al. (2012) showed that the mean score of organizational commitments of healthcare workers were high and positive [20]. In a study by Khodadadei et al. (2018) showed that the organizational commitment was moderate in nurses [21]. A study by Hadizadeh Talasaz et al. (2014) showed that the mean score of organizational commitment in midwives working in health centers was 76.40 ± 10.06 and in midwives working in maternity hospitals was 75.61 ± 11.09. In a study by Hadizadeh Talasaz et al. (2014), organizational commitment was low and very low and a small number had high organizational commitment [22]. A study by Siew et al. (2011) showed that 51% of nurses had high organizational commitment, 44% had moderate organizational commitment and 1% had low organizational commitment [23]. Since in this study, the healthcare workers had high and positive organizational commitments, it shows that they are very loyal and committed to their job to the organization. The authors of this article argue that religion may be one of the reasons for the high organizational commitments of health workers in Babol. The people of Iran are Muslims and have the religion of Islam. The religion of Islam emphasizes that if a person has made a commitment to another person such as the employer and the work environment, he or she should adhere to it and should not do less. Therefore, by strengthening Islamic knowledge in healthcare workers, we can see higher organizational commitments in them.

The mean of the highest scores in the organizational commitment questionnaire was related to satisfaction with the work in the health organization and related to feel honored to work for the health organization. The results of this section are expected to be useful for health managers because they should know whether the healthcare workers are proud of their job and duty that is in order to maintain the health of the people of the community. Therefore, managers must provide favorable conditions for healthcare workers to adapt to the work environment and solve their problems, because the satisfaction of them in health centers has a positive effect on the productivity and efficiency of the organization.

Chi-square showed that job position was significantly related to organizational commitments (*p =* 0.001) so that health groups had more organizational commitments. Healthcare workers serving in rural areas had higher organizational commitments (*p =* 0.002). In this study, health group had higher organizational commitments. This indicates that health group were loyal to the organization because the tasks of health group during the coronavirus pandemic were very difficult, including prevention, follow-up and screening, and they had to identify contaminated sources, equipment and surfaces, potential patients and carriers in very difficult conditions, in cities and villages, on impassable roads and paths.

According to the results of Tables 2 and 4, organizational and social commitments were higher in healthcare workers aged 20 to 30 years and lower in the elderly. The authors hypothesize that the reasons for low commitments in elderly workers could be the type of employment because elderly workers are formal employees and younger people are temporary employees. Formal staff have job security and are not easily fired, but temporary employees may be fired for a mistake or complaint. Therefore, young people try to do their job better with more organizational and social commitments.

In this study, none of the healthcare workers got into the category of low social commitments. A number of 24 healthcare workers (9.2%) were in the medium and 236 healthcare workers (90.8%) were in the high social commitment group. A study by Hassanian et al. (2017) showed that 72.6% of nurses had high social commitment [14]. In this study, the healthcare workers had high and positive social commitments. Social commitments can improve the performance of individuals and organizations, so strengthening them. This is necessary in all social, economic, cultural, and health-related organizations.

In the present study, he mean of the highest scores in the social commitment questionnaire regarding the right of people to have adequate health care services and regarding happiness in solving people’s problems during the coronavirus. In a study by Khalili et al. (2017) showed that humanitarian responsibility had a positive and significant relationship with the employees’ legal responsibility [18]. In a study by Asartamar et al. (2019) it was reported that there was the least effect between the components of social commitment, humanitarian responsibility and legal responsibility [10]. A study by Kim Chung et al. (2013) reported that employees’ social commitments were directly related to their job morale and motivation, and political and cultural factors were related to the social commitment of organizations [24].

## Conclusion

The results of this study showed that at the time of the coronavirus outbreak, the healthcare workers in Babol had very positive and high organizational and social commitments. As the world struggles with the coronavirus pandemic, employee and organizational productivity may decline due to the fear and anxiety of healthcare workers in various organizations. It is suggested that managers of health-related organizations, social, economic, and cultural organizations use the results of this study to identify factors affecting the organizational and social commitments of employees and strengthen them. It is recommended that staff social and organizational commitments should be increased by holding training classes virtually or through national and international media such as radio and television.

## Data Availability

The datasets used and analysed during the current study are available from the corresponding author upon reasonable request.

## Abbreviations

OCQ: Porter Organizational Commitment Questionnaire
COVID-19: Coronavirus disease 2019
CSR: Carroll’s social responsibility
Spss: Statistical package for social science
